# LDMOS Channel Thermometer Based on a Thermal Resistance Sensor for Balancing Temperature in Monolithic Power ICs

**DOI:** 10.3390/s17061397

**Published:** 2017-06-15

**Authors:** Tingyou Lin, Yingchieh Ho, Chauchin Su

**Affiliations:** 1 Institute of Communications Engineering, National Chiao-Tung University, Hsinchu 30010, Taiwan; 2 Department of Electrical Engineering, National Dong-Hwa University, Hualien 97401, Taiwan; 3 Department of Electrical Engineering, National Chiao-Tung University, Hsinchu 30010, Taiwan; ccsu@mail.nctu.edu.tw

**Keywords:** monolithic power ICs, temperature sensor, oscillator, thermal balancing, power metal-oxide-semiconductor (MOS), calibration

## Abstract

This paper presents a method of thermal balancing for monolithic power integrated circuits (ICs). An on-chip temperature monitoring sensor that consists of a poly resistor strip in each of multiple parallel MOSFET banks is developed. A temperature-to-frequency converter (TFC) is proposed to quantize on-chip temperature. A pulse-width-modulation (PWM) methodology is developed to balance the channel temperature based on the quantization. The modulated PWM pulses control the hottest of metal-oxide-semiconductor field-effect transistor (MOSFET) bank to reduce its power dissipation and heat generation. A test chip with eight parallel MOSFET banks is fabricated in TSMC 0.25 μm HV BCD processes, and total area is 900 × 914 μm^2^. The maximal temperature variation among the eight banks can reduce to 2.8 °C by the proposed thermal balancing system from 9.5 °C with 1.5 W dissipation. As a result, our proposed system improves the lifetime of a power MOSFET by 20%.

## 1. Introduction

Monolithic power integrated circuits (ICs) are popular in small and medium power applications such as power modules for LED lighting and portable devices [1,2,3]. High-voltage (HV) switching metal-oxide-semiconductor field-effect transistor (MOSFETs) and low-voltage (LV) control circuits are integrated in a single chip for not only reducing the circuit footprint but also decreasing the ICs package cost. Both the static power dissipation and the power efficiency can be improved accordingly. However, thermal management and reliability are significant challenges [4,5,6,7]. Most monolithic power ICs use lateral double-diffused MOSFETs (LDMOSs) as the switching devices. They are inferior to discrete switches, such as vertical double-diffused MOSFETs (DMOSs) or integrated gate bipolar transistors (IGBT) in that they have larger turn-on resistances *R_on_* [8]. A larger *R_on_* implies greater power dissipation, *P_d_* = *I_L_^2^R_on_*. However, the temperature distribution is affected by many kinds of factors. As the number of transistors is more than 10,000 in a discrete power MOSFET IC, the transistor’s lifetime will decrease as the channel temperature exceeds 75 °C. Therefore, the on-chip channel temperature sensing is necessary to optimize the operations of power MOSFET.

**Figure 1:**
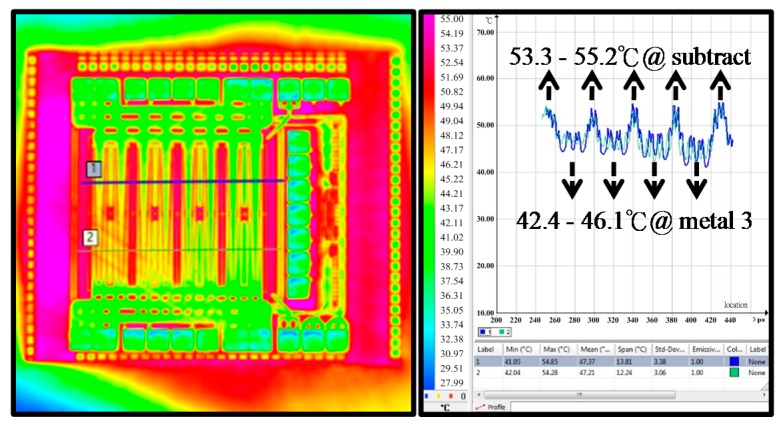
Infrated thermal photo of a power MOSFET.

A high-voltage power MOSFET that is constructed as a parallel MOSFET bank of multiple smaller transistors is called a large array device. The transistors are arranged in a sophisticated manner so that load current is uniformly distributed to them in parallel. Many layout styles, including multi-fingers, waffle, overlapping circular gate, wave, and diamond styles have been developed. However, the uniform distribution of the load current is still difficult to guarantee [9]. Figure 1 shows an infrared thermal photograph of a 45 V N-type power MOS with a power dissipation of 1 W. The temperature at the substrate ring is 53.3–55.2 °C, and that at the top metal above the transistor channel is 42.4–46.1 °C. However, both the temperatures are not the exact temperature in the transistor channel. Back-end layers could cause the infrared thermal method of temperature measuring to yield erroneous results owing to die surface condition and radiative transfer [10]. As a rule of thumb, every 10 °C increases in the channel temperature decreases the lifetime of semiconductor device in a half [11].

Thermal resistance model is a popular method to derive channel temperature from the case or the ambient temperatures, but it is an average value. The finite element (FE) thermal model simulates the thermal characterization of high-power modules by using RC network for thermal impedance [12]. It is effective to design heat dissipations in the presets, if the equivalent RC network is completely tracked. Some on-chip temperature sensors have been proposed to measure exact temperature from solid-state devices. These sensors are inherently sensitive to temperature. Diodes have been used in their analog counterparts because the yield voltage is a positive temperature coefficient due to reverse saturation current *I_S_* [13]. However, the diodes and analog to digital converter (ADC) are not suitable for the high-resolution power IC applications owing to their large layout size, nonlinearity characteristics, and large temperature variations in measurements. In [14], a diode is used and an ADC is applied to perform the readout. Moreover, the system requires the area overhead and has to work at over 100 °C. In some cases, bandgap-based voltage reference circuits are used as their voltages are proportional to absolute temperature (PTAT) [15]. A differential temperature sensor is used to determinate the efficiency of Radio frequency (RF) linear power amplifiers [16]. In addition, a voltage-to-frequency converter is used to increase the resolution and simplifies the calibration method. Apart from above, even in most digital ICs, ring oscillators as temperature sensors are used due to delay lines, which depend on device temperature [17,18,19,20,21,22,23]. As temperature sensors need to be embedded near the channel of power MOS, the ring oscillators and bandgap-based sensors are not suitable for MOS bank because of latch-up rule violation and the layout area in consideration. Temperature balancing technologies are common in the multi-core processor for controlling the performances of each core and further reducing and balancing the temperatures. In [24], the strategy of the equalizing temperature in integrated circuits has been reported to the optimum choice of the stabilized chip temperature. However, the technique is seldom used in power applications.

In this paper, a method of thermal balance with on-chip temperature monitoring is proposed for monolithic power ICs. The method identifies the hottest of MOSFET bank and reduces its power and thermal dissipation. The temperature-sensing device is an on-chip poly-silicon strip. A temperature-to-frequency converter (TFC) is developed to quantize an on-chip temperature as a digital code. For considering the chip area and balancing the control bits, the power MOSFET architecture can be divided into eight banks to monitor the channel temperature in the bank level. An average temperature monitor is proposed because the temperature will be saturated to a DC value after the chip working for a while. Based on the output of the temperature sensor, the hottest device can be identified. The equivalent duty cycle can thus be adjusted.

The rest of the paper is organized as follows. Section 2 develops an on-chip temperature monitoring technique. Section 3 introduces the pulse-width-modulation (PWM) methodology for temperature balancing. Section 4 details the corresponding circuit implementation and measurements made on a 0.25 μm 1P3M test chip. Finally, Section 5 draws conclusions.

## 2. Materials and Methods Proposed On-Chip Temperature Monitoring

**Figure 2:**
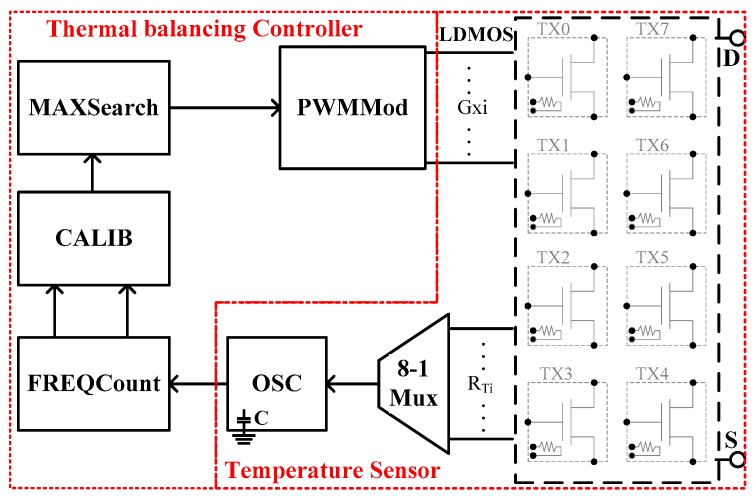
Proposed thermal balancing architecture.

Figure 2 presents the system block diagram of the proposed on-chip thermal balancing architecture, which is composed of two parts. The right part includes a temperature sensor with eight MOSFET banks in a parallel, an 8-1 Mux, and an RC oscillator (OSC). Each MOSFET bank has an individual gate control signal and a temperature sensing poly resistor. The 8-1 Mux is used to select one resistor for sensing the local temperatures and is connected to the OSC. The left part includes the thermal balancing controller, which is used to perform thermal balancing, which is verified by a field programmable gate array (FPGA) and the principle is introduced in Section 3.

**Figure 3:**
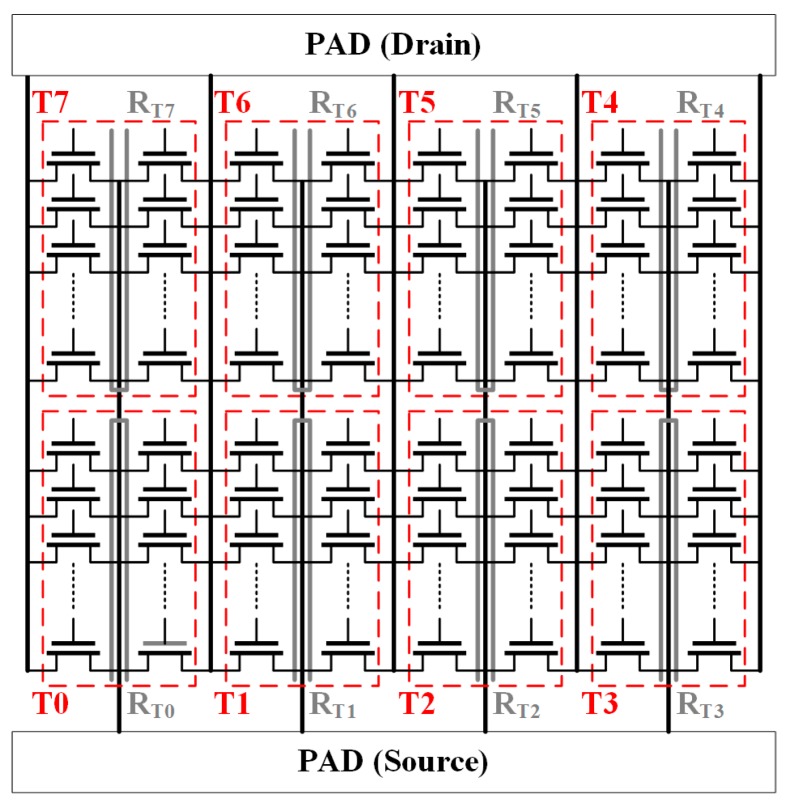
Equivalent circuit of eight MOSFET banks with poly-silicon strips as temperature sensing devices.

**Figure 4:**
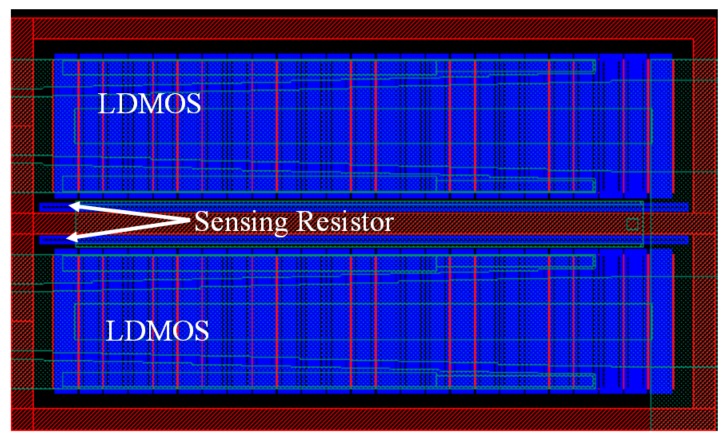
Layout of each MOSFET bank with a temperature sensor.

The sensing device in our temperature sensor is a poly-silicon strip, which can be placed next to the drain/source of the MOSFET bank under consideration. Figure 3 presents the eight parallel banks. A poly-silicon strip (thick gray line) is embedded in the middle of each MOSFET bank. Each bank has a total width of 5000 μm (50 μm × 50 fingers × 2 blocks), and the layout of poly and oxide diffusion (OD) layers as shown in Figure 4. The embedded sensors use to measure each of the MOSFET banks and notify the temperature variation within neighboring cells.

The change in resistance of the integrated resistor with temperature is

(1) $$R\left(T\right)=R_{O}\times \left(1+TC1\times \Delta T+TC2\cdot \Delta T^{2}\right)$$

where *R_O_* is the resistance at room temperature, *TC1* and *TC2* are the first and second-order temperature coefficients, and Δ*T* is the change in temperature. TSMC design guideline indicates that Δ*R/R_O_* equal to −9% in a poly resistor for any change in temperature (Δ*T*) varies from 25 to 150 °C, whereas *TC2* has a small effect on resistance because *TC1* >> *TC2* (larger than two orders). The simulated temperature coefficient is

(2) $$TC1_{S}=\frac{\Delta R/R_{0}}{\Delta T}=\frac{-9\%}{150-25}=-0.072\%/℃.$$

**Figure 5:**
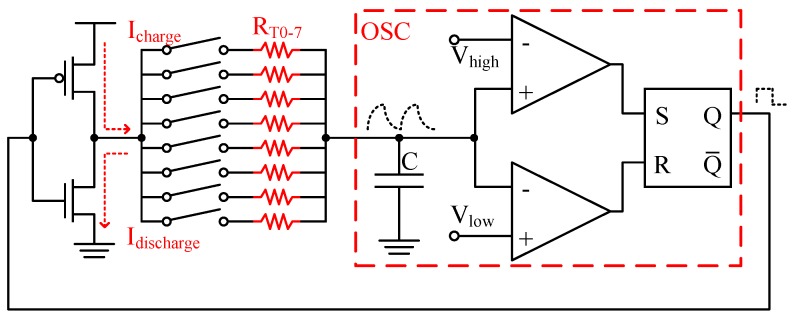
Proposed TFC circuit.

Figure 5 shows the detail circuitry of the proposed TFC, which is a stable multi-vibrator where *R_T_* is a poly resistor of the poly-silicon temperature sensor and a capacitor (*C*) is added to adjust the frequency of the OSC. The OSC includes two rail-to-rail comparators and an set-reset (SR) latch. The oscillation period is as follows.

(3) $$\tau =\tau_{1}+\tau_{2}=R_{T}\times C\times \left(ln\frac{V_{dd}-V_{low}}{V_{dd}-V_{high}}+ln\frac{V_{high}}{V_{low}}\right).$$

For example, *V_dd_* = 2.5 V, *V_low_* = 0.15 V, and *V_high_* = 2.35 V and set, so *τ* = 5.5 × *R_T_* × *C* and *f* = 1/*τ*. The design parameters are set as follows. The poly-silicon strip is 2 μm wide and 480 μm long with an equivalent resistance of 41 kΩ and *C* is 8 pF. According to Equation (3), the oscillation period is 1.8 μs, and the oscillation frequency is 554 kHz.

Based on Equation (1), the frequency or the period can be obtained to determine *R_T_* if *C* is known. The measuring time determines the accuracy of temperatures, which can be derived as follows. Suppose that *τ_min_* and *τ_max_* are the minimal and maximal periods, according to Equation (1), at maximal and minimal operating temperatures, *T_max_* and *T_min_*. *N* is the resolution and the number of temperature levels between *T_max_* and *T_min_*. The measuring time *τ_meas_* must satisfy

(4) $$\frac{\tau_{meas}}{\tau_{min}}-\frac{\tau_{meas}}{\tau_{max}}\geq N.$$

(5) $$\tau_{meas}\geq \frac{\tau_{max}\cdot \tau_{min}}{\tau_{max}-\tau_{min}}\times N.$$

If *τ_meas_* = (1 ± *x*)·*τ_o_*, then *τ_o_* is the period in which the average of oscillation period is determined, and *x* is the maximal deviation from the average,

(6) $$\tau_{meas}\geq \frac{\tau_{o}}{2x}\times N.$$

For example, if *τ_o_* is 1 μs and *x* is 5%, then *τ_min_* = 0.95 μs and *τ_max_* = 1.05 μs. For a given resolution, say *N* is equal to 1000, and for a fixed value of *τ_meas_* = 10 ms, the maximal count of *τ_meas_*/*τ_min_* is 10,526, and the minimal count of *τ_meas_*/*τ_max_* is 9524. In this case, a 14 bit counter is required to count maximal and minimal value of measured frequency.

Unfortunately, *C*, *R_T_*, and *x* are difficult to predict precisely. Process variation is another critical challenge in temperature sensing. As shown in Figure 5, all of the temperature sensing resistors (*R_T0–7_*) share the same capacitor, comparators, SR latch, and inverter in the TFC circuit. Ideally, the oscillation frequency is determined only by *R_T_*. Suppose that MOSFET banks *M_i_* and *M_j_* have temperature *T_i_* and *T_j_*, respectively. With a negative temperature coefficient, once the oscillation frequency *f_i_* is higher than *f_j_*, then *T_i_* is higher than *T_j_*. Since the oscillation frequency depends on the sensing resistor *R_T_* and capacitor *C*, a target of the frequency range can be designed with the size of the poly-silicon strip and the capacitor. The sensing resistor is designed related large as compared to the parasitic resistance of interconnections and TFC switches. As a result, we assumed that *R_T_* >> *R_P_*. In addition, the temperature sensitivity of the sensing resistor is larger than the parasitic resistance of the capacitor. The frequency change is mainly related to the sensing resistor. Thus, the variation of parasitic resistances and capacitor can be ignored in the TFC circuit. However, process variation and circuit mismatch affect the results from metal routing paths, switching devices, and sensing resistors, even at a fixed temperature. Consequently, a mechanism to calibrate process variation and mismatches is developed.

## 3. Calibrations and Thermal Balancing Methodology

**Figure 6:**
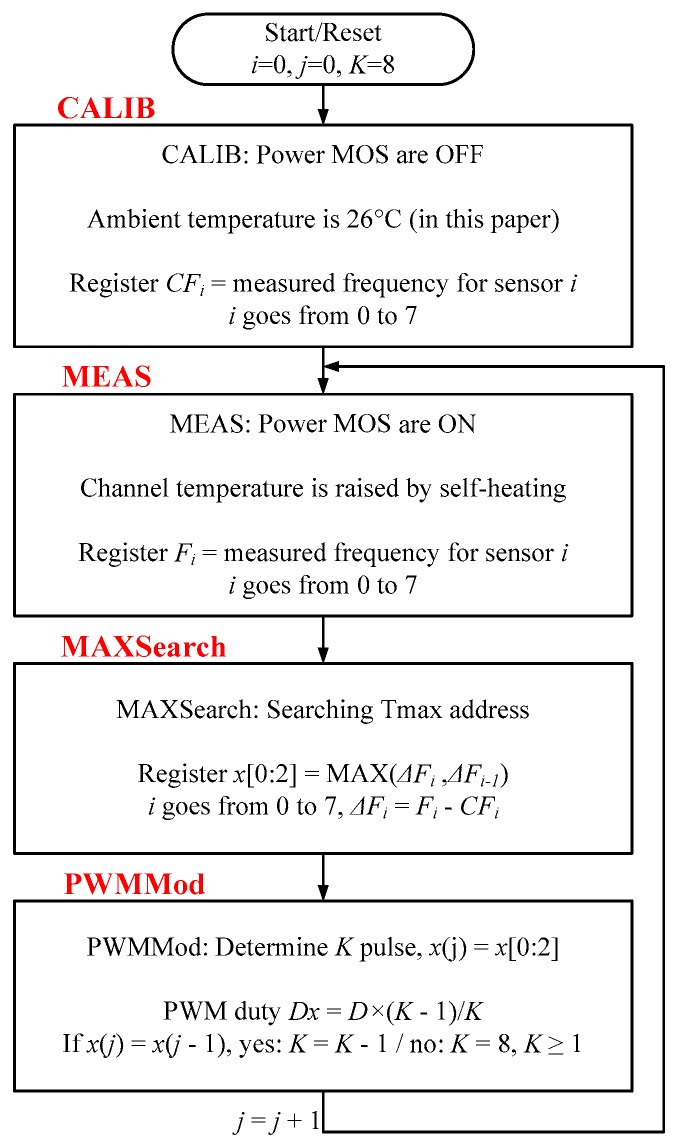
Flowchart of proposed thermal balancing mechanism.

Since the poly resistor determines the frequency of the TFC, as a function of temperature, the power MOS operation with self-heating can balances the channel temperature in each bank by pulse-width-modulation (PWM) methodology. *F_i_* is the total number of frequencies recorded by frequency count module (FREQCount) and calibrate module (CALIB) is a calibration module that overcomes accuracy problems related to process variation and circuit mismatch. MAXSearch finds the MOSFET bank with the highest temperature, whereas PWMMod reduces the number of PWM pulse of the hottest bank to lower down its temperature. Figure 6 shows the flowchart of the proposed thermal balancing method. The above loop will keep iterating whenever power and PWM signal are provided.

**Figure 7:**
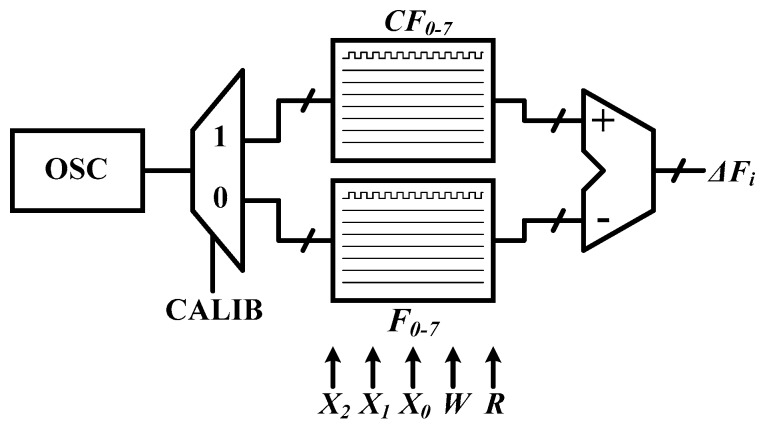
Block diagram of the calibration module.

The circuit mismatch is suppressed by the TFC circuit with an oscillator and eight sensing resistors, and a subtractor of the calibration method is expected to cancel the process mismatch. In the calibration module, Δ*F_i_ is* the number of changed frequencies, recorded and compared with *F_i_*, whereas Δ*F_i_* is defined as Δ*F_i_* = *F_i_* − *CF_i_*. *CF_i_* is the number of frequencies of the *i*^th^ MOSFET bank at the calibration time when power devices are not functioning. Figure 7 displays the block diagram of the calibration module in Figure 2. It consists of two register files, *CF_0–7_* and *F_0–7_*. *CF_0–7_* store the number of calibration frequencies. At the beginning of CALIB = 1, the power MOS is turned off, and the sensing resistors are sequentially selected. The frequency of the TFC is recorded in *CF_i_*. In the operation mode and measuring (MEAS) mode of CALIB = 0, the frequencies are counted, and the number is recorded as *F_i_*. Finally, *CF_i_* is subtracted from *F_i_*, yielding Δ*F_i_* for comparison of the channel temperatures. For example, the total number of frequencies is stored in a counting time of 1 ms, the oscillator through a sensing resistor, *R_T2_*, working at *T* = 25 °C and *T* = 100 °C (after heating). The results are subtracted, *F_0,100 °C_* – *CF_0,25 °C_*, yielding the temperature level in transistor bank 2 (TX2).

**Figure 8:**
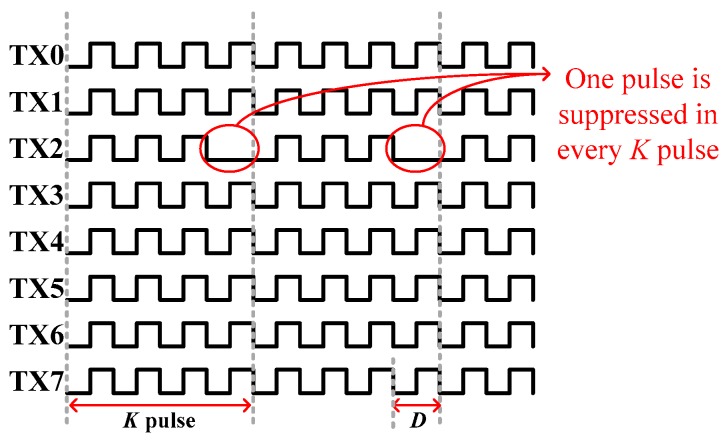
Gate control scheme of PWM modification to each MOSFET banks for thermal balancing.

The hottest bank is defined as the one with the largest Δ*F_i_*. After the hottest device has been identified, a PWM modification scheme for that bank is employed. A PWM signal is a repetitive pulse stream that periodically turns on devices. To reduce the temperature of the hottest bank, one pulse is suppressed in every K pulse, as shown in Figure 8. In Figure 8, TX2 is assumed to be the hottest bank. The gate control for TX2 has an effective duty cycle of 3/4 × *D* if one out of four pulses is suppressed. Therefore, the power dissipation of TX2 is 3/4, reducing the temperature.

However, decreasing the effective duty cycle for TX2 will reduce the overall power output. In a closed loop system, the feedback mechanism is activated to increase the duty cycle to maintain the overall power output level. Suppose that *N* numbers of MOSFET banks are in parallel and the original and the final duty cycle are *D_0_* and *D_1_*, respectively. The overall duty cycles of all *N* devices, or total output current, must be equal to each other before and after PWM modification. Therefore,

(7) $$N\times D_{0}=\frac{\left(K-1\right)}{K}\times D_{1}+\left(N-1\right)\times D_{1}.$$

(8) $$D_{1}=\frac{N}{\left(N-1/K\right)}\times D_{0}.$$

The left- and right-hand sides of Equation (7) are the accumulated duty of all devices. According to Equation (8), the adjusted duty cycle is greater than the original. Consider for example, *K* = 4, *N* = 8, and *D_0_* = 0.25; the adjusted duty cycle *D_1_* = 0.258, increasing by 0.8% after PWM modification. Accordingly, TX2 has an effective duty cycle of 0.194 (3/4 × 0.258), which is 78% of the original 0.25. The remaining seven banks share the suppressed pulse of TX2, with an increase in the adjusted duty cycle of 0.8%.

## 4. Experimental Results and Discussion

**Figure 9:**
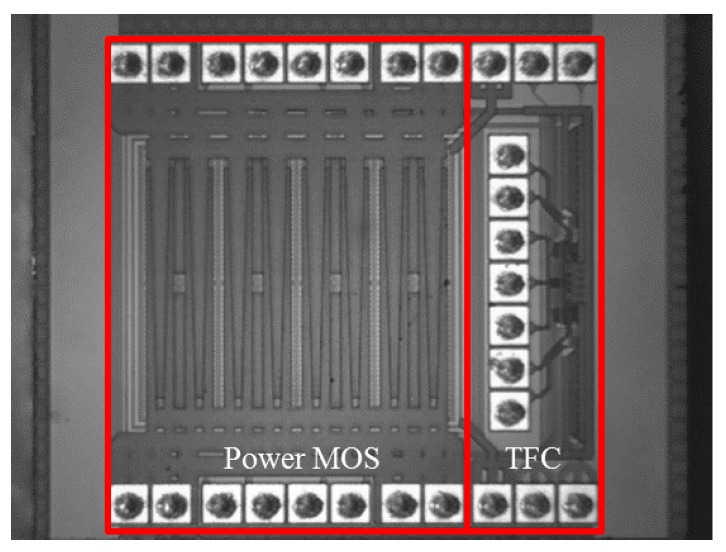
Die photograph.

To demonstrate the feasibility of the proposed methodology, a test power chip and a test board are used. An on-chip temperature sensor is implemented using TSMC 0.25 μm BCD processes. Figure 9 shows the die photograph of the proposed test chip. The test chip includes eight LDMOS with individual gate controls and a common source and drain, an 8-1MUX, and an OSC.

The experiment is composed of the following five steps. First, cad tools, R3D (Silicon Frontline, San Jose, CA, USA) and ANSYS Multiphysics (ANSYS, Canonsburg, PA, USA) are used to simulate the thermal effects of power MOS transistors and the distribution of temperature in MOSFET banks. Second, the post-layout simulation is performed to verify the TFC conversion mechanism. Third, the chip is put into a lab oven to validate and calibrate the temperature coefficients according to the RC OSC. Fourth, the CALIB and MEAS modes are conducted to count the oscillation frequencies with 0–1.5 W power dissipation. Finally, the PWMMod reduces the effective duty cycle for the hottest device. It confirms that the proposed algorithm reduces the peak temperature and any kind of variation in temperature for eight MOSFET banks.

### 4.1. Power and Thermal Simulation

**Figure 10:**
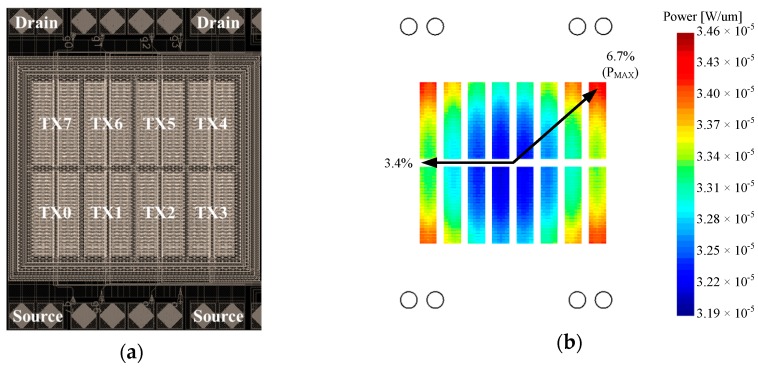

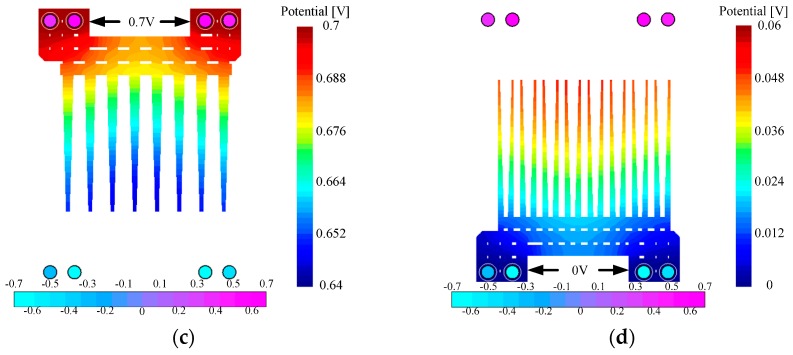
R3D and ANSYS simulations of: (**a**) the power MOS layout; (**b**) the power density at channel; (**c**) the drain side potential; (**d**) the source side potential at the top metal layer.

**Figure 11:**
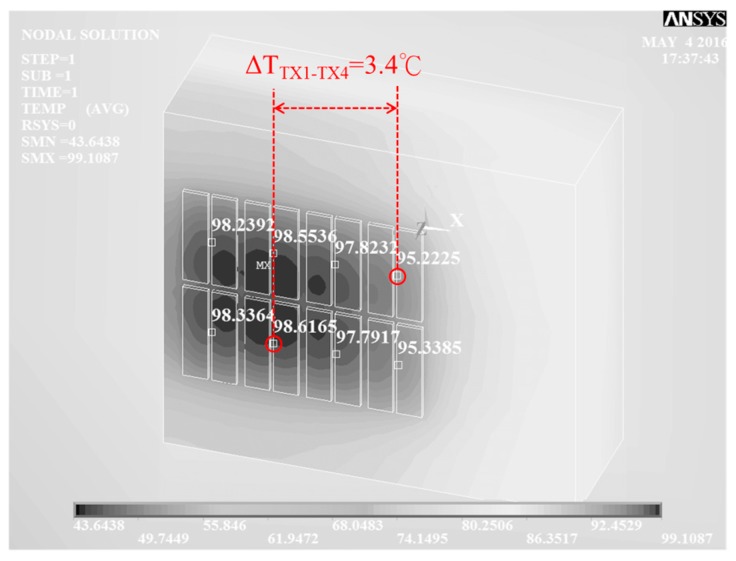
ANSYS simulations of banks temperature results.

Based on the power MOS layout in Figure 10a, Figure 10(b–d) show the power MOS simulated IR drop, power dissipation, and temperature distribution, obtained using R3D, based on a power dissipation of 1.5 W (*I_D_* = 2.14 A, *V_DS_* = 0.7 V at 25 °C). They show power density in W/μm (Figure 10b), IR drops of drain voltage distribution (Figure 10c), and source voltage distribution in the top metal layer (Figure 10d), and the overall temperature distribution in silicon (without metal consideration) is also shown. These figures indicate that the maximal power dissipation mismatch is 6.7%. The power dissipation is 1.5 W uniformly to eight parallel MOSFET banks without metal wiring. The distribution of power dissipation, obtained by R3D, is fed into ANSYS to conduct a thermal analysis. Figure 11 shows the results of ANSYS simulation related to the distribution of power dissipation results. The maximal temperature difference between the banks is 3.4 °C, where TX1 is the hottest bank and TX4 is the coolest bank. Figure 10 shows the hot spot with common-centroid distribution, which can be achieved by the symmetrical layout and the power devices placements theoretically. However, the temperature distribution is formed by many factors such as power dissipations, heat source locations, and thermal sink device areas [24]. In practical cases, the power MOSFET is operated under non-ideal conditions, such as turned-on uniformity, bonding wire consistency, and chip and package symmetry. Therefore, the hot spot is transferred to the left side of the chip in Figure 11 due to the location of the power MOS.

Based on the 3D simulation results, we can predict the expected temperature imbalances to determine the sensitivity and resolution of the implemented sensors as shown in Figure 11. The simulation shows the temperature distribution based on the boundary conditions of specific power dissipations, volumes, and material properties. Moreover, conditions of the power dissipations of banks, the places of heat sources, and the sizes of thermal sink components can be changed to make the analyses more accurate, whereas the routing metal, bonding wire, packaging material, and testing Printed circuit board (PCB) are the part of the thermal sink devices. However, these boundary condition factors would result in the difficulties of the analyses. Moreover, these factors would not add new data regarding to the expected temperature imbalances.

### 4.2. Temperature Coefficient Extraction

To verify the RC OSC and the temperature coefficient of the sensing poly-resistors (*R_T_*), the test chip is placed in a temperature-controlled lab oven. The oscillation frequency is 508.2 kHz when *V_high_* = 2.35 V and *V_low_* = 0.15 V. Table 1 shows the total number of frequencies *F_i_* and frequencies increased Δ*F_i_*, measured by using the FPGA board. The first row presents the set temperatures. The second row presents the measurements of frequencies obtained by FREQCount and CALIB from FPGA. The third row is the calibration result (Δ*F_T_* = *F_T_* − *CF_T_*). The temperature coefficient is derived as follows.

(9) $$TC=\frac{F_{100}-F_{25}}{\Delta T}=\frac{45995-43524}{100-25}=33/℃.$$

**Figure 12:**
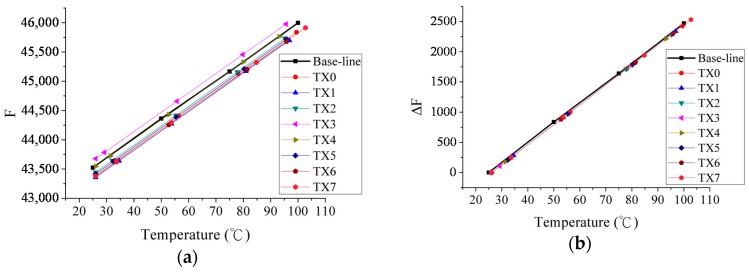
Frequency of the eight temperature sensors plotted as a function of temperature: (**a**) Before calibration; (**b**) after calibration.

From Equations (2) to (3), it can be inferred that the frequency depends on the resistance as follows: (*F_i_* − *CF_i_*)/*CF_i_* ∝ (*R_Ti_* − *R_Oi_*)/*R_Oi_*. The measured temperature coefficient *TC1_M_* is −0.0716%/°C and the simulated temperature coefficient *TC1_S_* is −0.072%/°C. Since the measurement time is 83.34 ms, the desired *TC* is 396 Hz/°C (*TC*/83.34 ms). The derived *TC* is a linear approximation of an exponential RC OSC. Figure 12 shows a plot of temperatures in the horizontal axis and frequency in the vertical axis, the frequency of the eight temperature sensors as a function of temperature before and after the calibration. In Figure 12b, we can see the dispersion between sensors and linearities, fitting the baseline of Table 1.

Based on these results, the frequencies offsets of the resistance mismatches can be obtained in Figure 12a. The temperature coefficient TC can be estimated according to the calibration in pre-measurement process. Therefore, the absolute temperatures can be obtained by the calibration. The probability errors of the measurements are about 1% of the resistance mismatch for relative errors and ±30% of the resistor tolerance for absolute errors. Despite the research on the maximal temperature address of the MOSFET bank, the absolute temperature information is not necessary. The calibration *ΔF* compensates the frequency error by the subtraction method.

### 4.3. Experiments and Discussion

**Figure 13:**
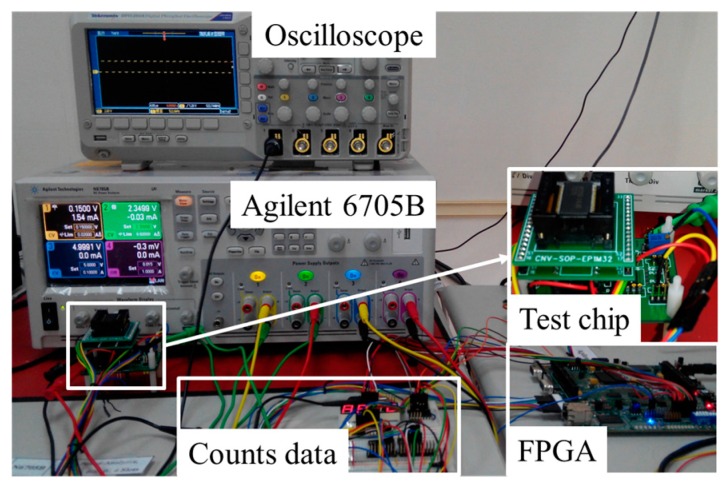
Photograph of measurement apparatus.

Figure 13 shows the experiment that involved a channel thermometer. In this experiment, measurements were made by forcing the continuously dissipated power into the power MOS using an Agilent N6705B source meter. The FPGA board recorded the total number of frequencies. Table 2 lists the measured temperatures based on *TC* = 33/°C; the counts after calibration are shown in parentheses. For comparison with each bank, the maximal and minimal temperatures are 102.7 °C and 93.1 °C. ANSYS simulations yield corresponding values of 98.55 °C and 95.34 °C. The maximal temperature difference is 9.6 °C (TX7 vs. TX4). The proposed method is highly accurate because it involved calibration using a lab oven.

### 4.4. Thermal Balancing by PWM Modification

**Figure 14:**
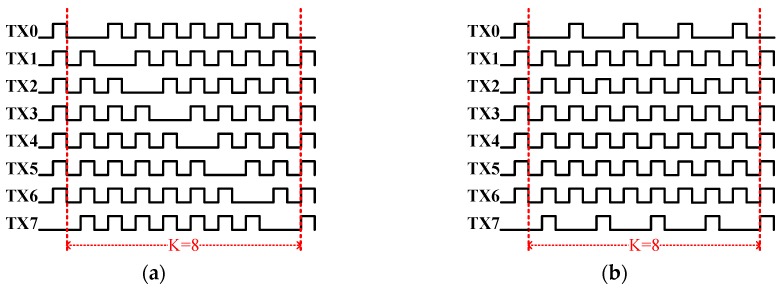
Each gate signal in the lateral double-diffused MOSFET (LDMOS) (**a**) before modification and (**b**) after modification.

To verify the effectiveness of the thermal balancing method, the following experiment was conducted. The LDMOS was connected to a 6705B DC Power Analyzer (Keysight, Santa Rosa, CA, USA) to force a dissipation of *P_D_* = 0−1.5W, and each gate was controlled by a 50% duty signal. To provide one pulse room for PWM modification, one pulse out of eight was reduced, as shown in Figure 14a. This will allow another seven pulses to turn on. Table 3 lists temperature measurement results before thermal balancing. Since the MOS power dissipated at 0–0.5 W, the measurement results for each bank in Table 3 were close to each other and a significant temperature difference of 9.5 °C at *P_D_* = 1.5 W.

**Figure 15:**
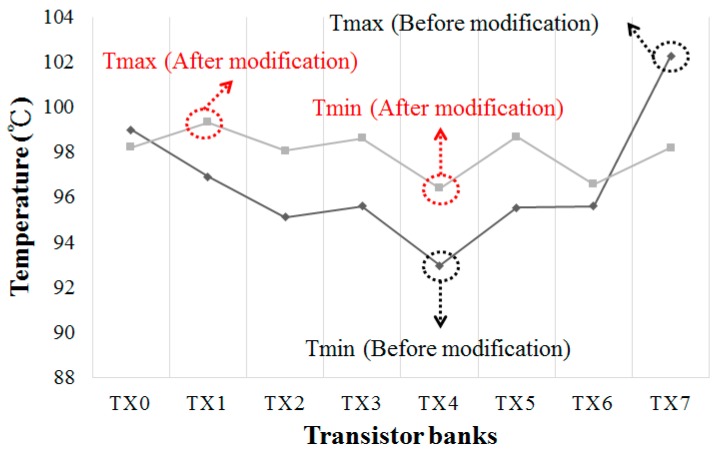
Photograph of measurement apparatus.

Since TX7 and TX0 were the hottest banks at *P_D_* = 1.5 W, their effective duty cycle was reduced to 1/2 × 50% from 7/8 × 50% by PWMMod (as one of every two pulses was suppressed). To maintain the overall duty cycle, the duties for coolest banks TX1–6 are increased to 8/8 × 50% (without suppressed). Figure 14b plots each gate signal after PWM modification. The original active duty cycles are all 7/8 × 50% as shown in Figure 14a. Moreover, for maintaining the equal electrical characteristics of the power MOS, seven out of eight banks of the power MOS are turned on during each cycle. Figure 15 shows the temperature profiles with and without PWM modification. The temperatures of TX0 and TX7 are reduced, whereas the temperatures of TX1-6 are increased because their duty cycles are different from TX0 and TX7. The PWMMod yield the PWM signals without suppressing the duty cycle for 8/8 × 50% from 7/8 × 50% of TX1–6. Therefore, the original temperature difference of 9.5 °C is reduced to 2.8 °C (99.2–96.4 °C).

### 4.5. Lifetime Improvement

This paper shows that the equalized temperature in eight banks of power MOS. Based on the studies in [25,26], the lifetime can be predicted according to the temperature acceleration model as follows.

(10) $$AF_{T}=\frac{\tau_{OP}}{\tau_{S}}=e^{\frac{E_{a}}{k}\;\times \;\left(\frac{1}{T_{OP}}-\frac{1}{T_{S}}\right)}$$

where *AF_T_* is the temperature acceleration factor. *E_a_* is the activation energy, which is assuming to 0.7 eV for the silicon junction defect, and *k* is the Boltzmann constant is 8.617 × 10^−5^ eV. A lifetime test for reliability requires $\tau_{OP}$ = 1000 h (0.34 y) at an operating channel temperature of *T_OP_* = 150 °C. Herein, *T_S_* is the stress temperature, and $\tau_{S}$ is the obtained lifetime. Table 4 presents the estimated lifetime based on Figure 15. The worse bank determines. Therefore, the lifetime of the power MOS can be improved by 20%, while the balancing method changes the worst bank from TX7 to TX1.

## 5. Conclusions

This paper proposes a thermal balancing method for monolithic power ICs. The method yields the exact chip temperature and heat distribution, which can thus be changed to improve the lifetime of the power MOS in the test chip. According to the readings of a real-time LDMOS junction thermometer, the thermal balancing method modulates individual gate PWM control and modifies the operating time of each MOSFET banks of the power MOS. The proposal includes a calibration mechanism that mitigates process variation and layout mismatches. A test chip with eight parallel MOSFET banks was fabricated by using the TSMC 0.25 μm HV BCD process. The balancing mechanism was developed through the FPGA board. The experimental results inferred that, under a power dissipation of 1.5 W, the maximal temperature variation among the eight banks was 9.5 °C without modification and 2.8 °C with modification. The full chip lifetimes of the power MOS are determined by the maximal temperature of the MOSFET bank. This paper verified that the temperature of the hottest bank was reduced from 102.4 °C (TX7) to 99.3 °C (TX1), which improves the overall lifetime by 20%. Since a large active die area increases temperature difference, the lifetime is proportional to the area of power MOS. According to the temperature acceleration model, the lifetime can be further improved in practical cases.

## Figures and Tables

**Table 1:** Calibration the total number of frequency versus temperature.

	25 °C	50 °C	75 °C	100 °C
Measured *F*	43,524	44,362	45,165	45,995
Measured *ΔF*	0	838	1641	2471

**Table 2:** Calibration the total number of frequency versus temperature.

Bank	0 W	0.1 W	0.5 W	1 W	1.5 W
TX0	26.0°C (0)	33.2°C (237)	56.3 °C (1001)	78.1 °C (1719)	99.5 °C (2424)
TX1	26.0°C (0)	34.5°C (282)	53.7°C (915)	80.9 °C (1812)	96.9 °C (2338)
TX2	26.0°C (0)	32.1°C (202)	55.4°C (970)	77.8 °C (1711)	95.2 °C (2284)
TX3	26.0°C (0)	29.2°C (106)	55.7°C (981)	79.8 °C (1776)	95.6 °C (2298)
TX4	26.0°C (0)	31.4°C (177)	52.8°C (883)	79.9 °C (1779)	93.1 °C (2215)
TX5	26.0°C (0)	32.5°C (214)	55.4°C (971)	80.4 °C (1794)	95.8 °C (2302)
TX6	26.0°C (0)	33.5°C (248)	52.7°C (882)	81.4 °C (1829)	95.8 °C (2303)
TX7	26.0°C (0)	33.6°C (252)	53.7°C (914)	84.8 °C (1941)	102.7 °C (2532)

**Table 3:** Measurement results of temperature at 7/8 × 50%.

Bank	0 W 0 A	0.1 W 0.29 A	0.5 W 0.63 A	1 W 0.87 A	1.5 W 1.03 A
TX0	26.0 °C	32.7 °C	55.9 °C	77.6 °C	99.0 °C
TX1	26.0 °C	34.4 °C	53.6 °C	80.8 °C	96.7 °C
TX2	26.0 °C	31.9 °C	55.2 °C	77.6 °C	95.0 °C
TX3	26.0 °C	34.2 °C	55.9 °C	80.0 °C	95.8 °C
TX4	26.0 °C	31.1 °C	52.5 °C	79.7 °C	92.9 °C
TX5	26.0 °C	32.1 °C	55.0 °C	80.0 °C	95.4 °C
TX6	26.0 °C	33.1 °C	52.3 °C	81.0 °C	95.4 °C
TX7	26.0 °C	33.3 °C	53.4 °C	84.5 °C	102.4 °C

**Table 4:** Lifetime estimation at 1.5 W power dissipation.

Bank	Before	Lifetime (Before)	After	Lifetime (After)
TX0	99.0 °C	4.73 y	98.2 °C	4.96 y
TX1	96.7 °C	5.42 y	99.3 °C	4.65 y
TX2	95.0 °C	6.00 y	98.1 °C	4.99 y
TX3	95.8 °C	5.72 y	98.6 °C	4.84 y
TX4	92.9 °C	6.81 y	96.4 °C	5.52 y
TX5	95.4 °C	5.86 y	98.7 °C	4.81 y
TX6	95.4 °C	5.86 y	96.4 °C	5.52 y
TX7	102.4 °C	3.88 y	98.2 °C	4.96 y
